# Iridium-based double perovskites for efficient water oxidation in acid media

**DOI:** 10.1038/ncomms12363

**Published:** 2016-08-08

**Authors:** Oscar Diaz-Morales, Stefan Raaijman, Ruud Kortlever, Patricia J. Kooyman, Tim Wezendonk, Jorge Gascon, W. T. Fu, Marc T. M. Koper

**Affiliations:** 1 Leiden Institute of Chemistry, Leiden University, PO Box 9502, Leiden 2300 RA, The Netherlands; 2 ChemE, Faculty of Applied Sciences, Delft University of Technology, Julianalaan 136, Delft 2628 BL, The Netherlands; 3 Department of Chemical Engineering, University of Cape Town, Private Bag X3, Rondebosch 7701, South Africa

## Abstract

The development of active, cost-effective and stable oxygen-evolving catalysts is one of the major challenges for solar-to-fuel conversion towards sustainable energy generation. Iridium oxide exhibits the best available compromise between catalytic activity and stability in acid media, but it is prohibitively expensive for large-scale applications. Therefore, preparing oxygen-evolving catalysts with lower amounts of the scarce but active and stable iridium is an attractive avenue to overcome this economical constraint. Here we report on a class of oxygen-evolving catalysts based on iridium double perovskites which contain 32 wt% less iridium than IrO_2_ and yet exhibit a more than threefold higher activity in acid media. According to recently suggested benchmarking criteria, the iridium double perovskites are the most active catalysts for oxygen evolution in acid media reported until now, to the best of our knowledge, and exhibit similar stability to IrO_2_.

## Abstract (web summary)

Iridium oxide is an active and stable catalyst for the oxygen evolution reaction, however Ir is very rare, making it unsuitable for large-scale application. Here the authors develop a class of Ir double perovskites containing less Ir than iridium oxide, but exhibiting 3-fold higher activity in acidic media.

## Graphical abstract

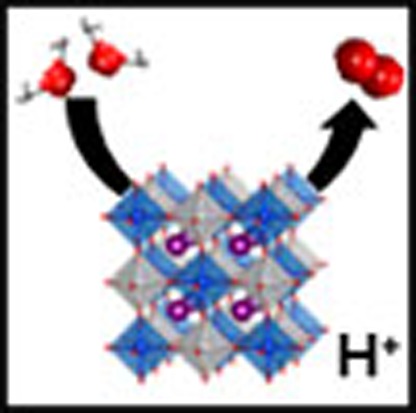

(Photo-)electrochemical generation of fuels is a promising way to store renewable energy. However, large-scale electrochemical fuel generation is limited by the slow kinetics of the anode reaction (oxygen evolution reaction, OER)^1^. An active, stable and cost-effective OER catalyst compatible with the working conditions required for high-rate fuel production in polymer electrolyte membrane (PEM) electrolysers is still lacking^2^. The best available compromise between OER activity and stability at the moment is iridium oxide IrO_2_ (refs 3, 4, 5), but it contains a large amount of iridium (one of the rarest elements on earth), making IrO_2_ unsuitable for large-scale applications. Consequently, the development of OER catalysts with smaller amounts of iridium metal is a requirement towards cost-effective multi-MW solar fuel generation using PEM electrolysers, especially in combination with iridium recycling^6^,^7^.

Complex iridium-based oxides (for example, perovskites, pyrochlores and fluorite-like compounds) are good alternatives to reduce the usage of the noble metal in the development of active and stable OER catalysts, by diluting iridium in a framework of less expensive materials. This alternative was explored by Kortenaar *et al*.^8^, who reported 12 iridium-based compounds with different crystal structures that exhibit from moderate to high OER catalytic activity in strong alkaline media (45% KOH). More recently, the group of Suntivich^9^ reported that the SrIrO_3_ simple perovskite exhibits higher OER activity than iridium oxide in alkaline media (0.1 M KOH).

Since iridium-based OER catalysts are well suited to work in the acid environment of PEM electrolysers, this work explores the versatility of complex oxides to develop a class of catalysts for the OER in acid media. We report a family of catalysts based on iridium double perovskites, which contain three times less iridium and yet exhibit a more than threefold higher activity per cm^2^ of real surface area (see Methods for details) for OER in acid media compared with IrO_2_, with similar stability. We show that these compounds are the most active catalysts for OER in acid media reported to date, to the best of our knowledge, according to recently suggested benchmarking criteria^10^.

## Results

### OER activity of the iridium-based double perovskites

**Figure 1:**
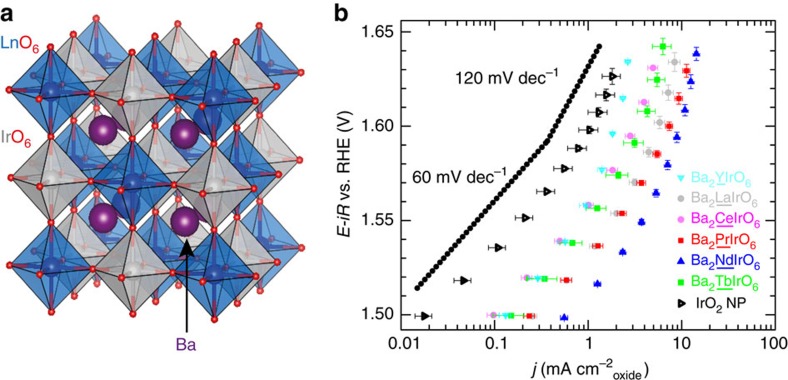
Crystal structure and OER activity of the double perovskites. (**a**) Crystal structure of a generic Ba_2_MIrO_6_ DP. (**b**) OER activity in 0.1 M HClO_4_ of Ba_2_MIrO_6_, M=Y, La, Ce, Pr, Nd, Tb, compared with the benchmark activity of IrO_2_ nanoparticles; the dotted lines show Tafel slopes of 60 and 120 mV per decade, to guide the eye. Measurements performed under steady-state conditions. The values in the plot are the average of three independent measurements and the error bars correspond to the s.d.

Double perovskites (DPs) are compounds with the generic formula *A*_2_*BB′*O_6_, with *A* denoting a large cation and *B* and *B′* smaller cations. The crystal structure of the ideal DP is depicted schematically in Fig. 1a (ref. 11). A wide range of compounds can be prepared by different combinations of *A*, *B* and *B′* cations, which allows fine tuning of the DPs. The activity (measured as the current density) towards OER in 0.1 M HClO_4_ (pH=1) was studied for Ba_2_MIrO_6_ DPs, with M=Y, La, Ce, Pr, Nd and Tb. The electrodes were prepared by drop-casting an ethanol-based ink of the DPs on the gold disk of a rotating ring-disk assembly and the electrochemical experiments were carried out under rotating conditions (see Methods for further details). The structure of the Ir DPs was verified by powder X-ray diffraction (XRD) and the patterns obtained agree with those reported in the literature^12^,^13^,^14^ (Supplementary Fig. 1). Figure 1b summarizes the measured catalytic activities in the form of Tafel plots and compares the activity with IrO_2_ nanoparticles that have been reported as the activity benchmark for OER in acid media^10^. The OER activity of the IrO_2_ nanoparticles compares well with the values reported previously for similar nanoparticles^10^,^15^.

All Ir DPs show a more than threefold higher activity per cm^2^ of real surface area towards OER compared with the benchmarking IrO_2_ nanoparticles. The surface area was calculated from pseudocapacitance measurements (see Methods for details). The OER activity depends on the cation in the B-site, in the order Ce≈Tb≈Y<La≈Pr<Nd. We note that we compare the intrinsic activity of the Ir DPs and the IrO_2_ nanoparticles on the basis of the electrochemically active surface area. This intrinsic activity may be influenced by particle size^16^. Our Ir DPs are rather large (∼1 μm, see below) so it is to be expected that further activity adjustments may result from developing synthesis procedures yielding smaller particles. In addition to particle size, pH is an important parameter in activity comparisons. While Fig. 1b applies to pH=1 as we are primarily interested in the OER activity in acid media, it is well known that many oxides active for OER exhibit strongly pH-dependent reactivities^17^,^18^.

Both the IrO_2_ nanoparticles and the iridium-based DPs show two values for the Tafel slope, ca. 60 mV per decade between 1.5 and 1.6 V and 120 mV per decade above 1.6 V (numerical values are given in Supplementary Table 1). This feature has been reported previously for IrO_2_ and was explained by a change in the mechanism for OH^−^ adsorption, which is thought to be the rate-determining step for OER on IrO_2_ (refs 19, 20). We note, however, that the Tafel slope measured at high potentials for Ba_2_YIrO_6_ (196+23 mV per decade) markedly deviates from the values for the other Ir DPs (ca. 120 mV per decade).

We also measured the Faradaic efficiency of Ba_2_PrIrO_6_ towards OER in 0.1 M HClO_4_ in a rotating ring-disk electrode (RRDE) configuration (Supplementary Fig. 2). It shows that Ba_2_PrIrO_6_ has an efficiency towards water oxidation higher than 90% at 1.50 V (*η*=0.27 V). The decrease in Faradaic efficiency with more anodic electrode potential is explained by the large current for OER above 1.6 V, leading to the formation of oxygen bubbles that reduce the detection efficiency on the ring.

### Stability of the Ir DPs under OER conditions

**Figure 2:**
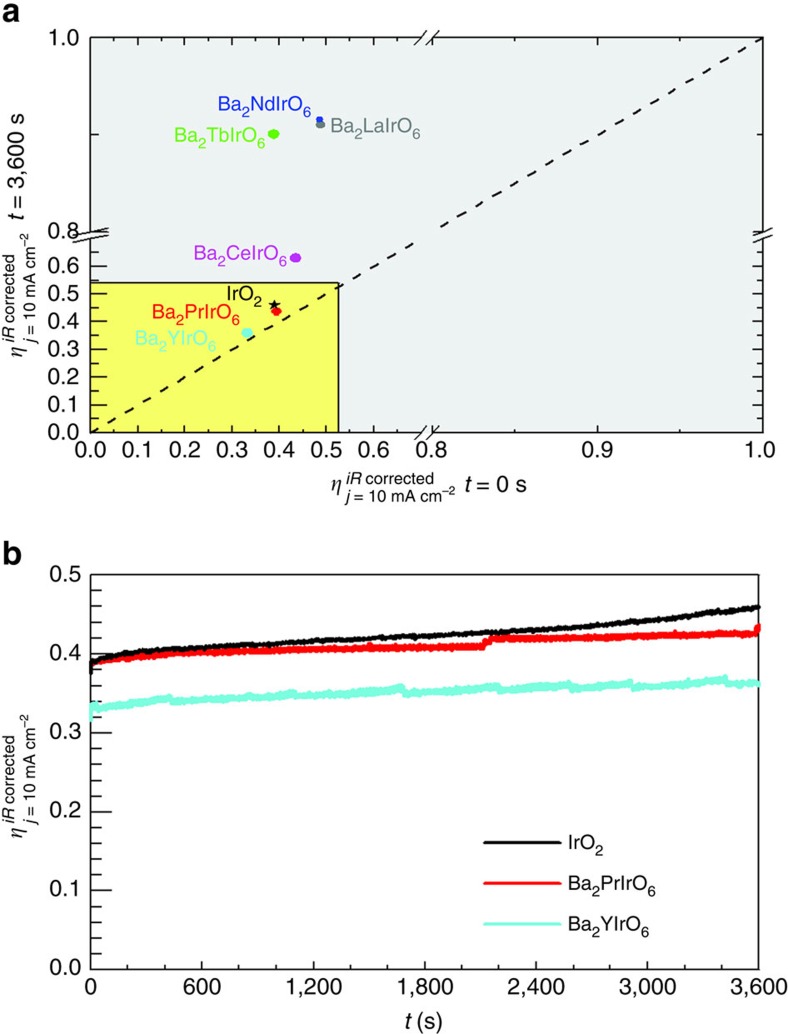
Stability of the double perovskites under OER conditions. (**a**) OER overpotential measured on the Ir DPs after 1 h of galvanostatic electrolysis plotted as a function of the initial overpotential. (**b**) Evolution of the OER overpotential on Ba_2_PrIrO_6_, Ba_2_YIrO_6_ DPs and IrO_2_ nanoparticles during 1 h of galvanostatic electrolysis.

The electrochemical stability of the Ir DPs was evaluated by galvanostatic electrolysis, in a manner similar to the approach reported by the JCAP group^10^. Figure 2a summarizes the results obtained from these experiments. The stability of IrO_2_ nanoparticles is presented for comparison. The diagonal dashed line represents the expected response of a stable catalyst.

The results in Fig. 2a show that Ba_2_PrIrO_6_ and Ba_2_YIrO_6_ DPs are electrochemically stable on the time scale of 1 h, operating at 10 mA cm^−2^, and they show a very similar stability to IrO_2_ under the same conditions, which is the state-of-the-art catalyst for OER in acid. The electrochemical stability of these compounds is illustrated by a plot of the Ohmic-drop-corrected overpotential as a function of time during the galvanostatic electrolysis (Fig. 2b). Ba_2_PrIrO_6_ and Ba_2_YIrO_6_ in fact show better stability in terms of overpotential than the IrO_2_ catalyst. The La, Nd and Tb-containing DPs are also very active towards OER in acid media according to the JCAP benchmarking, but they lose their activity after 1 h of galvanostatic electrolysis (Fig. 2a). It should be realized that the JCAP stability test is not a long-term stability test as required for operation in a commercial device, demanding operation for at least 500 h at current densities higher than 1.6 A cm^−2^ (ref. 7). However, it provides a simple and fast method to estimate both the activity and stability of potentially promising OER catalysts without engineering-related considerations.

Due to the small amounts of material used in the OER experiments (ca. 2.5 μg), we could not obtain powder XRD after the catalytic reaction. To assess the structural stability of the Ir DPs, Ba_2_PrIrO_6_ was examined by transmission electron microscopy (TEM) and XRD after treatment for 48 h in harsh media, namely 0.1 M HClO_4_ and 0.1 M HClO_4_+4 M H_2_O_2_. The Ba_2_PrIrO_6_ compound was selected for these experiments because it was one of the compounds that showed to be electrochemically stable (Fig. 2). The open-circuit potentials of the Ba_2_PrIrO_6_ DP in 0.1 M HClO_4_ solution and in 0.1 M HClO_4_+4 M H_2_O_2_ solution were measured to be 1.3 and 1.1 V, respectively. Therefore, the characterization of the treated powder in these two solutions should give an indication of the stability of the DP in acid at the onset of the OER. The TEM images in Supplementary Fig. 3 show that the original powder sample of Ba_2_PrIrO_6_ DP consists of rather large particles (ca. 0.5–2 μm), consistent with the sharp lines in the XRD pattern (Supplementary Fig. 1). The particle size of the Ba_2_PrIrO_6_ did not change significantly after treatment in 0.1 M HClO_4_ or 0.1 M HClO_4_+4 M H_2_O_2_ (Supplementary Fig. 3b,c) and the XRD pattern (Supplementary Fig. 4) remains virtually the same. This shows that the crystal structure of the DP is preserved during the treatment. However, there is a larger fraction of smaller particles (particle size <200 nm) in the samples that were leached compared with the pristine compound (see Supplementary Fig. 3a in comparison with Supplementary Fig. 3b,c). This suggests that Ba_2_PrIrO_6_ either cleaves or partially dissolves during the treatment but this process is slow (Supplementary Fig. 4).

We also characterized the surface composition of the pristine and treated Ba_2_PrIrO_6_ DP samples by X-ray photoelectron spectroscopy (XPS). The results of this analysis are summarized in Supplementary Fig. 5 and Supplementary Table 2 in the SI. These data indicate that the surface of Ba_2_PrIrO_6_ is not stable on the acid and the oxidative treatment. XPS results show surface enrichment in iridium after both treatments of Ba_2_PrIrO_6_, whereas the barium surface contribution reduces ca. three- to fourfold with respect to the pristine compound. The surface apparently enriches in praseodymium on the oxidative treatment (0.1 M HClO_4_+4 M H_2_O_2_), but this is caused by the formation of a Pr_2_BaO_4_ passivation layer over the perovskite particles. For details, see the Supplementary Note 1. The XPS results show that the surface iridium sites contain a mixture of Ir(IV) and Ir(V), and become enriched in Ir(V) upon the leaching treatments (see Supplementary Fig. 6 and Supplementary Note 1).

The leaching of the components of the Ba_2_PrIrO_6_ DP during OER was also assessed by elemental analysis of the electrolyte after electrolysis experiments of 1 h at constant electrode potential (Supplementary Table 3). The post-experiment analysis indicates that ∼10 wt% of Ba and Pr leached out of the Ba_2_PrIrO_6_ DP in the potential region 1.45–1.55 V, however, a negligible amount of iridium dissolves (<1 wt%), consistent with the evidence from XPS.

### Role of the crystal structure on the OER activity of Ir DPs

**Figure 3:**
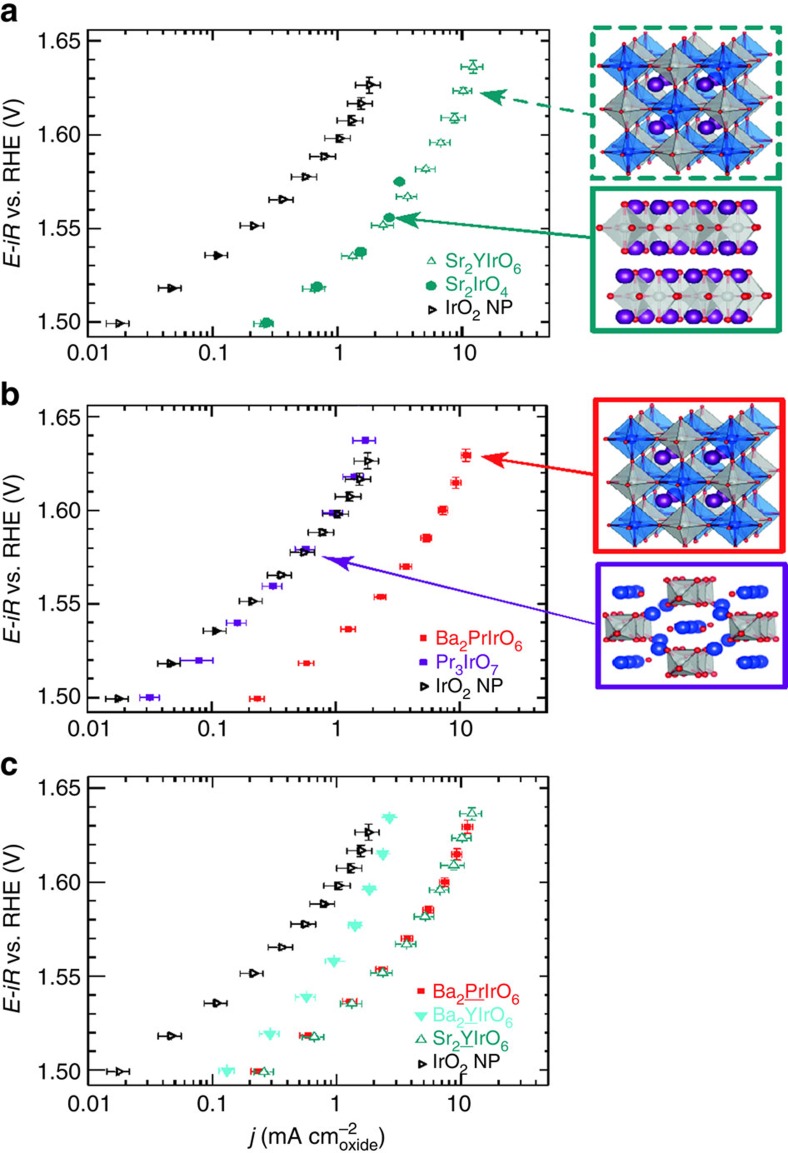
Structure sensitivity of the OER activity on the double perovskites. (**a**) OER activity in 0.1 M HClO_4_ of Sr_2_YIrO_6_ DP in comparison with Sr_2_IrO_4_. (**b**) OER activity in 0.1 M HClO_4_ of Ba_2_PrIrO_6_ DP in comparison with Pr_3_IrO_7_. (**c**) Effect of the *A* and *B′* cation substitution on the OER activity in 0.1 M HClO_4_ of the Ir DPs. The benchmark activity of IrO_2_ nanoparticles is shown for comparison. Measurements performed under steady-state conditions. The values in the plot are the average of three independent measurements and the error bars correspond to the s.d.

The OER activity sensitively depends on the crystal structure of the iridium-based catalyst. Figure 3a,b compares the activity of the Ba_2_PrIrO_6_ and Sr_2_YIrO_6_ DPs with Sr_2_IrO_4_ and Pr_3_IrO_7_ phases (the XRD and crystal structure of the Sr_2_IrO_4_ and Pr_3_IrO_7_ phases are shown in Supplementary Fig. 7 and the XRD of the Sr_2_YIrO_6_ is shown in Supplementary Fig. 1). The activity of Sr_2_IrO_4_ is quite similar to that of Sr_2_YIrO_6_ (Fig. 3a), whereas that of Pr_3_IrO_7_ is 10-fold lower than the Ba_2_PrIrO_6_, though comparable to the activity of the IrO_2_ nanoparticles (Fig. 3b). The structure of Sr_2_IrO_4_ and Pr_3_IrO_7_ also contain the corner-shared IrO_6_ octahedra, but differ from that of the DP in the network arrangement. The Sr_2_IrO_4_ forms two-dimensional (2D) perovskite-like layers^21^,^22^, separated by a rock salt layer of SrO (Supplementary Fig. 7a), whereas in Pr_3_IrO_7_ the IrO_6_ octahedra are linked by sharing the corner oxygen and form one-dimensional (1D) chains^23^ (Supplementary Fig. 7b). It is important to note that Sr_2_IrO_4_ is very unstable under the OER measuring conditions, and its activity abruptly decreases above 1.6 V due to decomposition, hence its OER activity in Fig. 3a is shown until ca. 1.58 V. This illustrates that the 2D arrangement of IrO_6_ octahedra is the minimum condition required for the enhanced OER activity but that the activity and stability of the Ir DPs is linked with the 3D perovskite arrangement.

## Discussion

Regarding the high activity measured for the Ir DPs, we postulate that the crystal lattice strain caused by the small lanthanide and yttrium cations in the *B′* site of the DPs may be related to the enhanced OER activity measured in these compounds. DFT calculations have shown that the substitution for small(er) *A*-site cations can decrease oxygen adsorption energies on simple perovskites (*ABO*_3_), due to the crystal strain^24^. Since IrO_2_ has been reported to bind oxygen too strongly for a truly optimal catalyst^25^,^26^, the lattice strain caused by substitution of smaller lanthanides or yttrium could weaken the oxygen adsorption energy and therefore improve the activity of the Ir DPs in comparison with IrO_2_. The effect of strain in the crystal lattice on OER activity of the Ir DPs was further confirmed by comparing the activity measured for Ba_2_YIrO_6_ and Sr_2_YIrO_6_ (Fig. 3c), showing the enhancing effect of the smaller strontium cation on the Ir DP with respect to its barium analogue, in agreement with the computational evidence reported for simple perovskites. The activity of Ba_2_CeIrO_6_ shows an additional effect related to the Ir^IV^ centres^12^,^13^; this increases the binding energy of adsorbates^24^ and may explain why this DP is the least active in the series. We speculate that the superior performance of these materials is due to the perovskite-like network optimizing the orbital interaction between the *B*,*B′* cations and O^2−^, and allowing adjustment of the *B–O* and *B′–O* bond distances to accommodate local charge changes. Since the binding of the OER intermediates requires the iridium centres to adjust their charges, the breathing mode displacement of oxygen in the perovskite-like network would favour lattice adjustments that do not exist in the rutile structure (IrO_2_) or in fluorite-like compounds. In addition, the observed surface leaching may also contribute to the enhancement of the OER activity of the perovskite surface by leaving behind highly active iridium centres.

In summary, we have reported a family of Ir DPs electrocatalysts with superior activity for water oxidation in acid media. Compared with IrO_2_, the state-of-the art benchmarking catalyst, these compounds contain 32 wt% less iridium and exhibit higher activity per real surface area and similar stability for the OER. Our results show that a 3D network of corner-shared octahedra is a necessary prerequisite for the activity enhancement of the Ir DPs as well as for their chemical stability under anodic working conditions. Our findings regarding the effect of the *B*-site cation on the catalysis towards OER of the Ir DPs suggest that the activity of these compounds might be further improved by carefully selecting the *A*- and *B*-site cations. Our strategy for thrifting iridium usage could also be extended to modulate the activity and stability of ruthenium-containing compounds, with a lower content of ruthenium compared with ruthenium oxide.

## Methods

### Chemicals

The water used to prepare solutions and clean glassware was deionized and ultrafiltrated with a Millipore Milli-Q system (resistivity=18.2 MΩ·cm and TOC<5 p.p.b.). All chemicals used in this work were of high purity (pro analysis grade or superior) and they were used without any further purification. The perchloric acid used for the electrolyte was from Merck (70–72%, EMSURE).

### Synthesis and characterization of the iridium-based catalysts

Ba_2_MIrO_6_ (M=La, Ce, Pr, Nd, Tb and Y) double perovskites were prepared from BaCO_3_, SrCO_3_, La_2_O_3_, CeO_2_, Pr_6_O_11_, Nd_2_O_3_, Tb_4_O_7_, Y_2_O_3_ and metallic iridium powder, respectively, in alumina crucibles, using the standard solid-state reactions described in literature^13^,^14^,^22^,^23^. All reactions were carried out in air and the products were furnace-cooled to room temperature. The powders were intermittently reground during the synthesis.

The suspension of iridium oxide nanoparticles was prepared by acid-catalysed condensation of [Ir(OH)_6_]^2−^ intermediate obtained from alkaline hydrolysis of Na_2_IrCl_6_ at 90 °C (ref. 27).

X-ray powder diffraction patterns were collected on a Philips X'Pert diffractometer, equipped with the X'Celerator, using Cu-Kα radiation in steps of 0.020°(2*θ*) with 10 s counting time in the range 10°<2*θ*<100°.

### Electrochemical experiments

Glassware was cleaned by boiling in a 3:1 mixture of concentrated sulfuric acid and nitric acid to remove organic contaminants, after which it was boiled five times in water. When not in use, the glassware was stored in a solution of 0.5 M H_2_SO_4_ and 1 g l^−1^ KMnO_4_. To clean the glassware from permanganate solution, it was rinsed thoroughly with water and then immersed in a diluted solution of H_2_SO_4_ and H_2_O_2_ to remove MnO_2_ particles, after which it was rinsed with water again and boiled five times in ultrapure water.

The electrochemical experiments were carried out at room temperature in a two-compartment electrochemical cell with the reference electrode separated by a Luggin capillary. Measurements were performed using a homemade rotating Pt ring–Au disk electrode (*φ*_disk_=4.6 mm) as working electrode, a gold spiral as counter electrode and a reversible hydrogen electrode (RHE) as reference electrode; a platinum wire was connected to the reference electrode through a capacitor of 10 μF, acting as a low-pass filter to reduce the noise in the low-current experiments. The electrochemical experiments were controlled with a potentiostat/galvanostat (PGSTAT12, Metrohm-Autolab) and they were performed either with cyclic voltammetry at 0.01 V s^−1^ or with potentiostatic steps of 0.02 V every 30 s. The latter procedure is referred to as steady-state measurements. Before and between experiments, the working electrode was polished with 0.3 and 0.05 μm alumina paste (Buehler Limited), subsequently it was sonicated in water for 5 min to remove polishing particles. The electrolyte was saturated with air prior to the experiments by bubbling for 20 min with compressed air, with the air first passed through a 6 M KOH washing solution. The RRDE experiments to measure the Faradaic efficiency were carried out with the electrolyte saturated with argon, which was purged through for at least 30 min prior to the experiment and kept passing above the solution during the measurement. For RRDE measurements, the Pt ring was kept at 0.45 V versus RHE while the disk with the catalyst loaded was scanned at 0.01 V s^−1^ in the potential range 1.25–1.75 V versus RHE. The efficiency (*η*) was calculated with equation (1), assuming that the oxygen reduction proceeds via four electron transfer, to produce water:


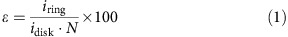


The collection factor (*N*) for oxygen was measured by using IrO_2_ as reference catalyst, and the value obtained was 0.199+0.001, which corresponds well with the value obtained from experiments with the redox couple [Fe(CN)_6_]^3−^/[Fe(CN)_6_]^4−^ (*N*=0.23); the small difference has been attributed to the non-ideal outward flow of O_2_ (ref. 15).

The catalysts were immobilized on the Au disk by drop-casting an ethanol-based ink of the oxides, using Na-exchanged neutral Nafion as binder^28^. The inks were prepared to yield the following final concentrations: 1 mg_oxide_/mL_ink_ and 0.7 mg_Nafion_/mL_ink_ (ref. 29). Prior to the preparation, the powders were ground in a mortar to eliminate big clusters. The appropriate amount of ink was drop-cast on the electrode to obtain 15 μg_oxide_ cm_disk_^−2^ of loading and this electrode was dried in vacuum; cm_disk_^2^ refers to the geometrical surface area of the Au disk.

The current densities reported in this work were calculated using the electrochemical surface area (addressed as real surface area) obtained from pseudo-capacitance measurements, assuming 60 μF cm^−2^ for the specific capacitance of the double layer^10^,^30^. This procedure of surface area determination was preferred over the Brunauer–Emmet–Teller (BET) method, based on the physical absorption of a gas on the oxide surface, because it allowed us to estimate the active electrochemical surface area in contrast to the surface area obtained from BET measurements^30^. The Tafel plots were corrected for the Ohmic resistance of the electrolyte, the resistance was measured by electrochemical impedance spectroscopy and by conductimetry^10^ and the value obtained was 24±1 Ω.

The amount of dissolved components from the Ba_2_PrIrO_6_ after electrochemical water oxidation experiments in 0.1 M HClO_4_ was by means of inductively coupled plasma atomic emission spectroscopy (ICP-AES). These experiments were performed potentiostatically (1 h electrolysis) and in non-rotating conditions, using 10 ml of electrolyte. The double perovskite catalyst was drop-cast on a gold disk (950 μg_oxide _cm_disk_^−2^ of Ba_2_PrIrO_6_), using an ethanol-based ink similar to the one described for the RRDE experiments but with higher perovskite content (30 mg_oxide_/mL_ink_). The electrolyte was analysed directly in a Varian Vista-MPX CCD Simultaneous ICP-AES. The fraction of dissolved metal (% M leached) was calculated as follows:


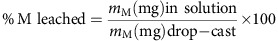


In this equation, *m*_M_ (mg)in solution was obtained from the ICP-AES analysis after the electrolysis experiment and *m*_M_ (mg)drop-cast corresponds to the mass of each component of the Ba_2_PrIrO_6_ double perovskite, calculated from its stoichiometry.

### Data availability

All relevant data are available from the authors upon request.

## Additional information

**How to cite this article:** Diaz-Morales, O. *et al*. Iridium-based double perovskites for efficient water oxidation in acid media. *Nat. Commun.* 7:12363 doi: 10.1038/ncomms12363 (2016).

## Supplementary Material

Supplementary Information Supplementary Figures 1-7, Supplementary Tables 1-3, Supplementary Note 1 and Supplementary References.

Peer Review File
