# Supplementary material for: Iridium-based double perovskites for efficient water oxidation in acid media
Source: Nat Commun. 2016 Aug 8;7:12363. doi: 10.1038/ncomms12363 (PMC4979062; doi:10.1038/ncomms12363)
Supplement: Supplementary Information — Supplementary Figures 1-7, Supplementary Tables 1-3, Supplementary Note 1 and Supplementary References. [file ncomms12363-s1.pdf]

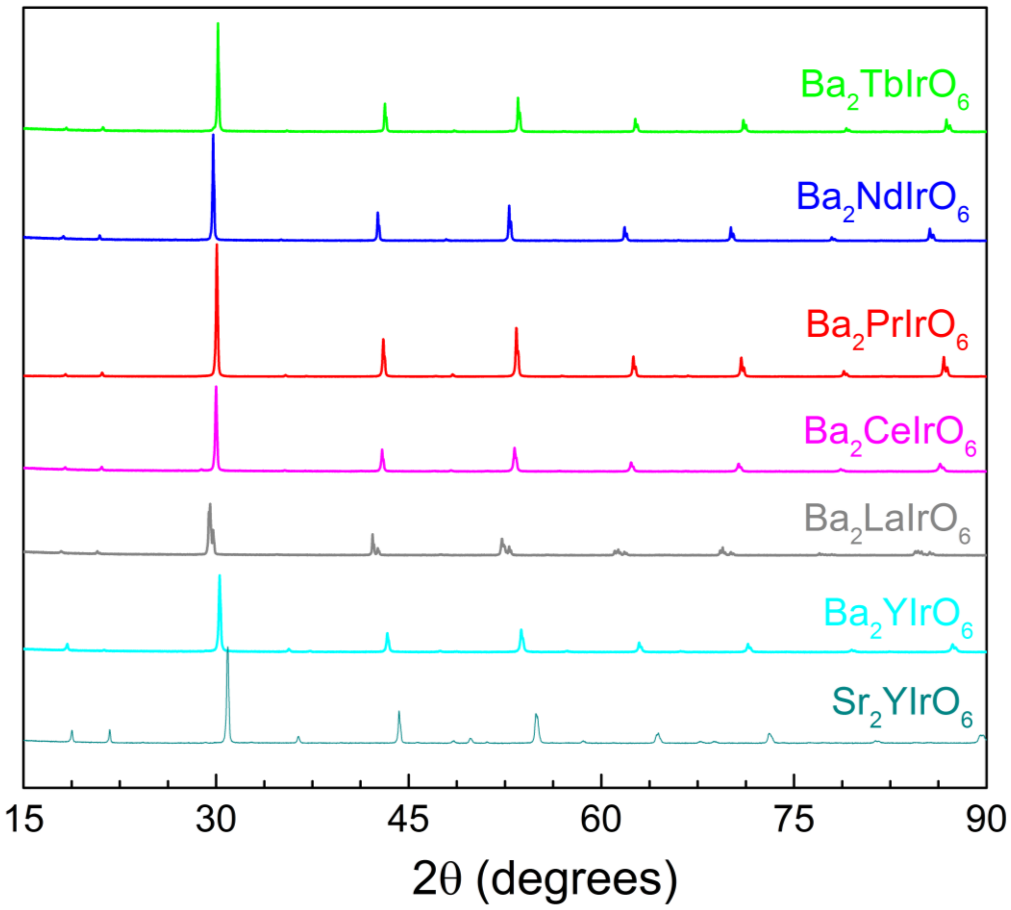

Supplementary Figure 1: Powder XRD for the iridium-based double perovskites.

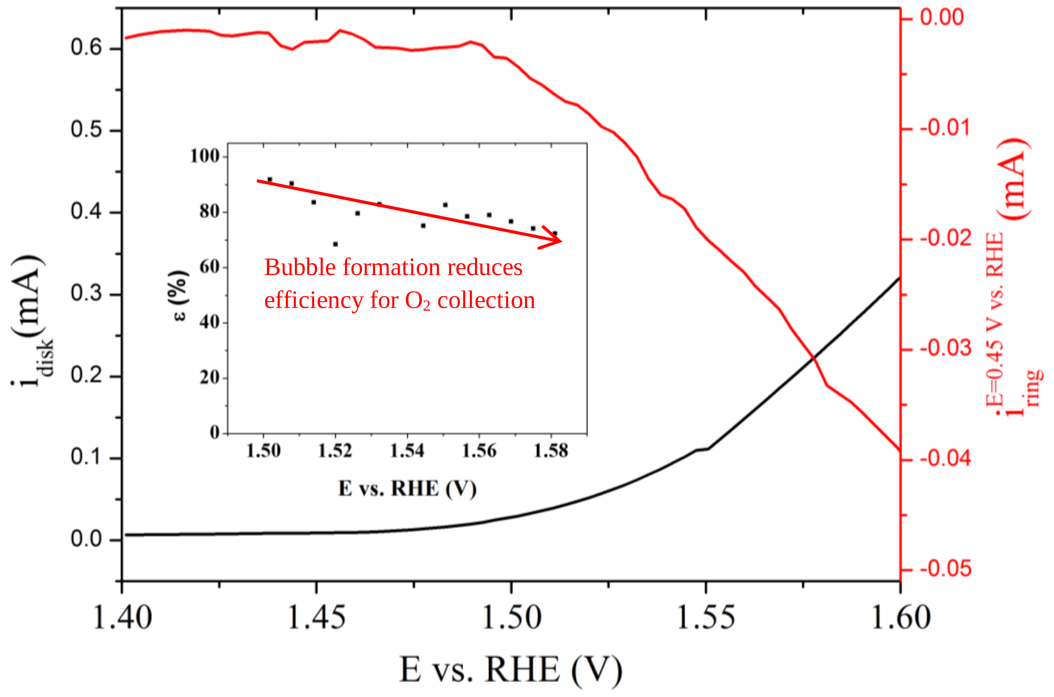

Supplementary Figure 2: RRDE measurement of the catalytic activity of Ba_2PrIrO_6 towards water splitting in 0.1 M $HClO_4$. Insert: Faradaic efficiency for water splitting.

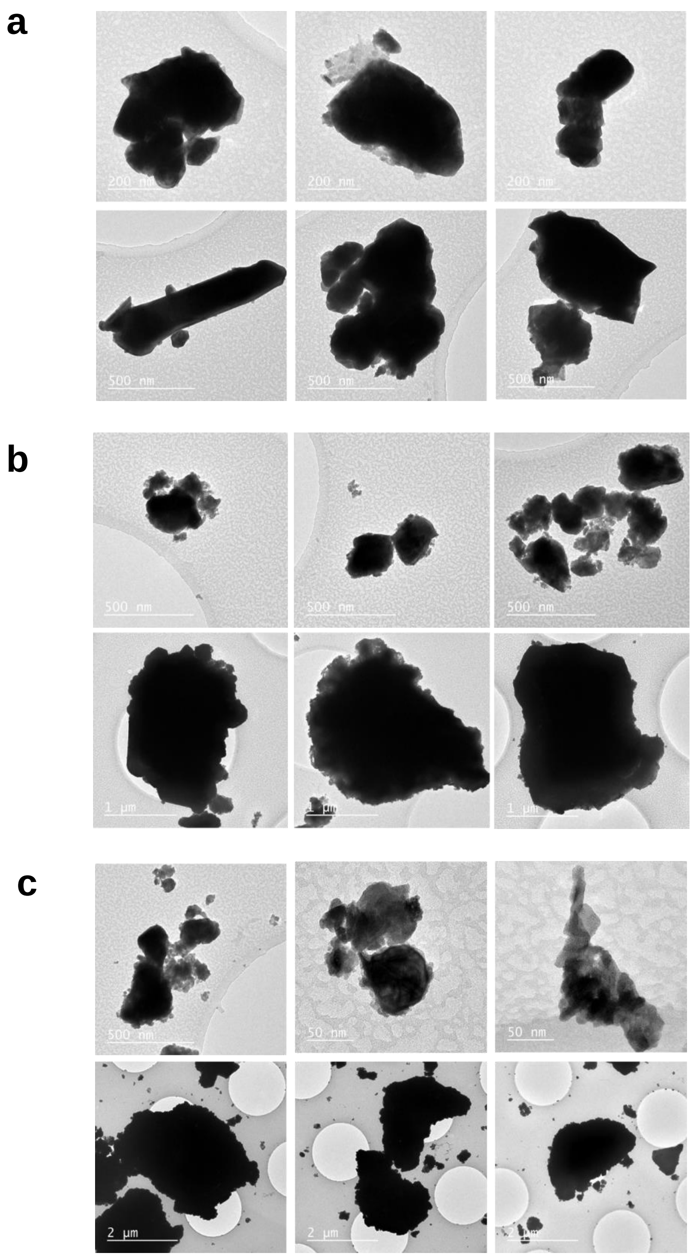

Supplementary Figure 3: TEM images of the pristine Ba_2PrIrO_6 DP (**a**), after 48 h leaching in $HClO_4$ 0.1 M (**b**) and $HClO_4$ 0.1 M + 4 M H_2O_2 (**c**).

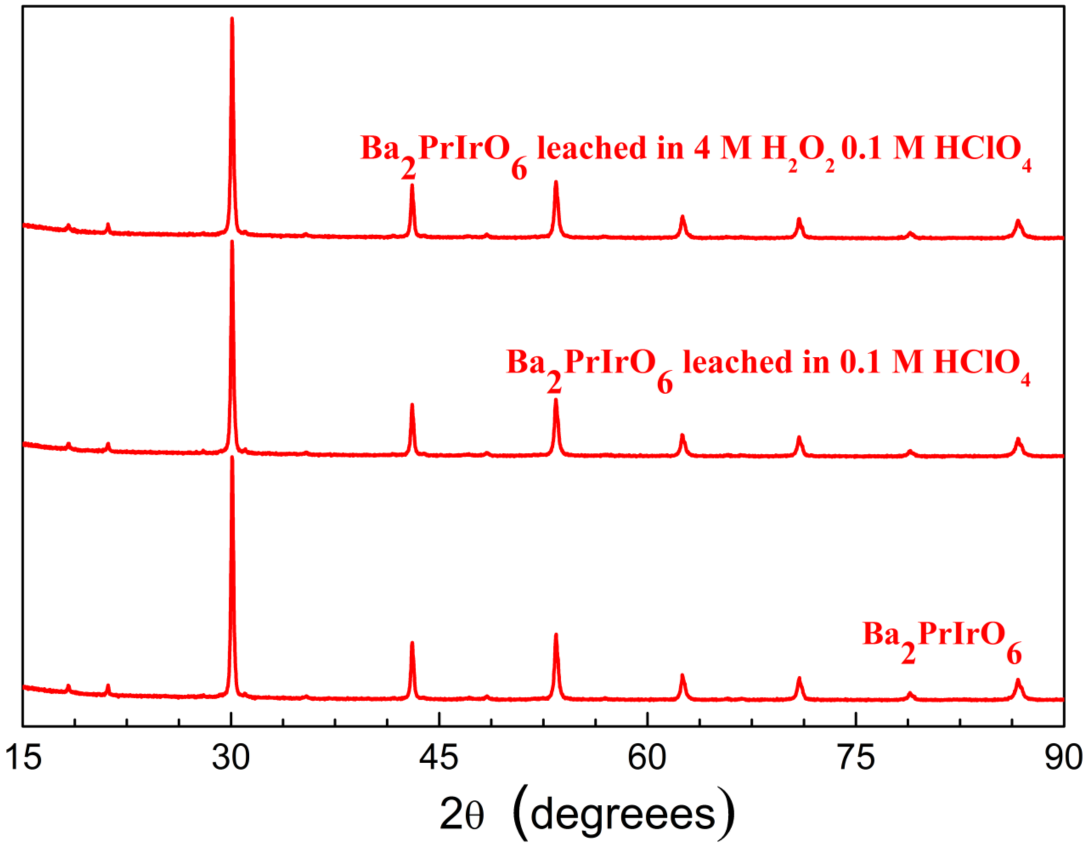

Supplementary Figure 4: Powder XRD of the Ba_2PrIrO_6 before and after the leaching treatments. XRD pattern of the Ba_2PrIrO_6 pristine sample is presented for comparison.

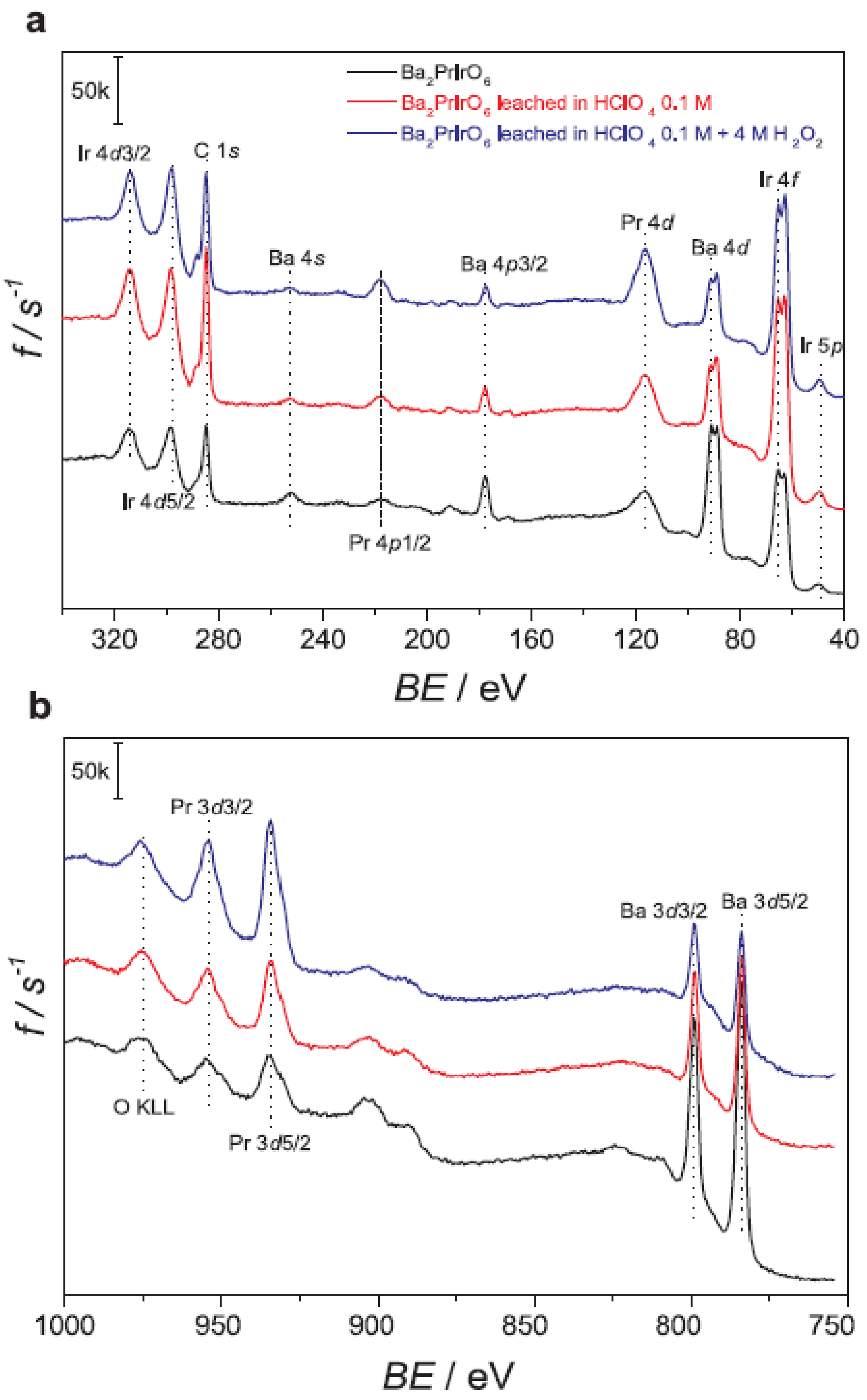

Supplementary Figure 5: XPS spectra of pristine a treated Ba_2PrIrO_6 double perovskite a) Binding energy region 750 – 1000 eV. b) Binding energy region 40 – 340 eV.

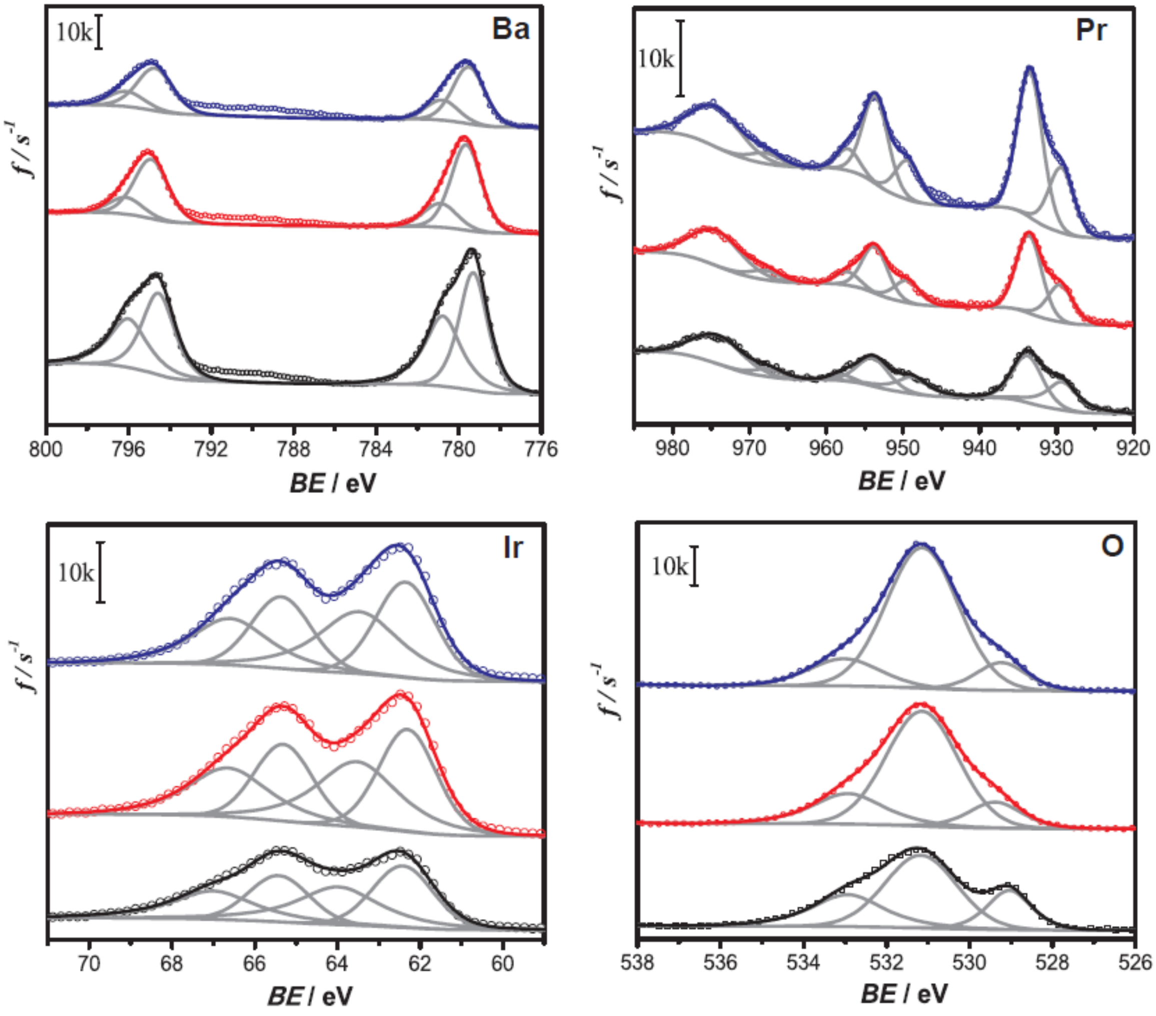

Supplementary Figure 6: Core-level XPS spectra (Ba3*d*, Pr3*d*, Ir4*f* and O1*s*) of Ba_2PrIrO_6 before and after the leaching treatments.

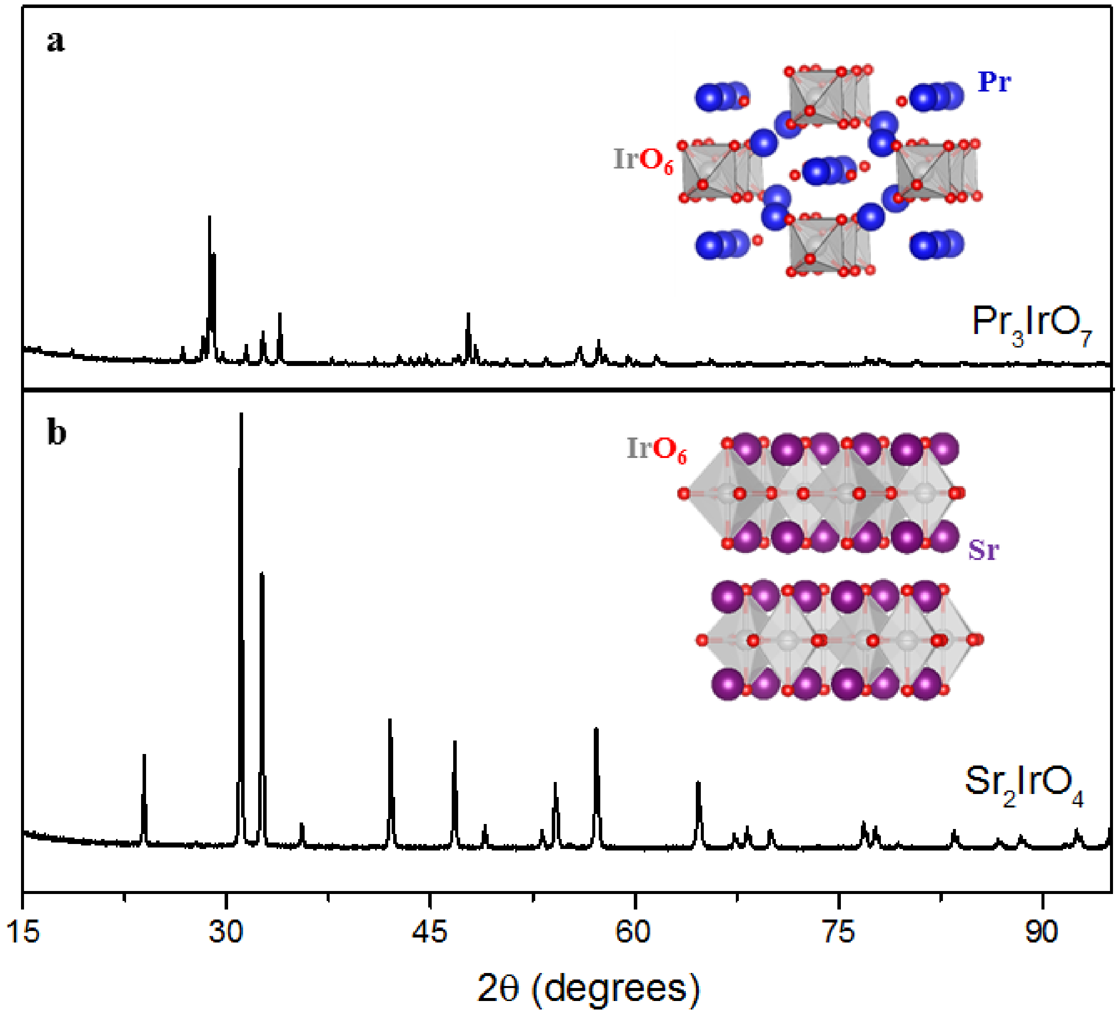

Supplementary Figure 7: a) Powder XRD for Sr2IrO4 Ruddlesden-Popper layered perovskite phase (b) Powder XRD for and for the Pr_3IrO_7 fluorite-like phase. The inserts in each panel of the figure show the crystal structure of each compound.

Supplementary Table 1: Tafel slope for the water splitting reaction in 0.1 M $HClO_4$ for IrO_2 and the iridium-based double perovskites. Currents were measured in steady-state conditions (Rotation rate: 1500 RPM). The values in the table are the average of three independent measurements and the error bars correspond to the standard deviation in the set of measurements.

	**Tafel slope (mV/dec) (E = 1.5-1.6 V vs. RHE)**	**Tafel slope (mV/dec) (E > 1.6 V vs. RHE)**
**IrO_2**	57 ± 1	115 ± 13
**Ba_2LaIrO_6**	59 ± 3	127 ± 10
**Ba_2CeIrO_6**	57 ± 7	121 ± 12
**Ba_2PrIrO_6**	54 ± 3	106 ± 17
**Ba_2NdIrO_6**	59 ± 4	136 ± 1
**Ba_2TbIrO_6**	61 ± 3	118 ± 9
**Ba_2YIrO_6**	67 ± 4	196 ± 23

Supplementary Table 2: Surface composition of Ba and Pr relative to Ir in the pristine Ba_2PrIO_6 and after the leaching treatments with 0.1 M $HClO_4$ and 0.1 M $HClO_4$ + 4 M H_2O_2, obtained from XPS analysis. The expected values were obtained from the chemical formula of the compound reported by Fu and Ijdo[1].

	**Ba: Ir**	**Pr:Ir**
**Expected values**	2.00	1.00
**Pristine Ba_2PrIrO_6**	2.46	0.97
**Ba_2PrIrO_6 leached in 0.1 M $HClO_4$**	0.85	0.77
**Ba_2PrIrO_6 leached in 0.1 M $HClO_4$ + 4 M H_2O_2**	0.60	1.39

Supplementary Table 3: Fraction of Ba, Pr and Ir dissolved from the Ba_2PrIrO_6 after 1 h of electrolysis at constant potential in $HClO_4$ 0.1 M.

E vs. RHE (V)	% Ba leached	% Ir leached	% Pr leached
**1.45**	12.7	0.6	9.5
**1.55**	14.2	0.8	11.3

Supplementary Note 1: X-ray photoelectron spectroscopy analysis of the leached Ir DPs

The surface composition of the pristine and treated Ba_2PrIrO_6 DP samples was characterized by means of X-ray photoelectron spectroscopy (XPS). The results of this analysis are summarized in Supplementary Figure 5 and in Supplementary Table 2.

It is clearly shown in Figure 3 that the pristine double perovskite contains a much larger contribution of Ba ($3d_{3/2}$, 794.9 eV; $3d_{5/2}$, 779.6 eV) relative to Pr ($3d_{3/2}$, 953.4 eV; $3d_{5/2}$, 933.0 eV) compared to the leached samples. The surface of the double perovskite enriches with praseodymium upon leaching with hydrogen peroxide and perchloric acid, which is evidences by the increase in the relative intensities of the Pr peaks in Figure 3. The intensity of the peaks attributed to iridium ($Ir4d_{3/2}$, 313.9; $Ir4d_{5/2}$, 298.1 eV and Ir4f, 63.8 eV) increases for the sample leached in 0.1 M HClO4, however, the oxidative treatment in 0.1 M $HClO_4$ + 4 M H_2O_2 slightly reduces the intensity of the iridium peaks, indicating that the noble metal started to leach.

The surface composition of the pristine and leached Ba_2PrIrO_6 samples is summarized in Supplementary Table 2. The results show that the relative atomic composition in the pristine double perovskite is very close to the expected values, indicating that the surface exhibits the same composition as the bulk of the double perovskite. Again, the results show the surface composition of the Ba_2PrIrO_6 enriches in iridium and praseodymium upon the leaching treatments, whereas the barium is depleted from the surface.

The change in the oxidation state of barium, praseodymium and iridium in the samples was analyzed using the high-resolution scans of the core-level in the XPS spectra. The oxidizing treatment did not eliminate or change the adventitious carbon signal, allowing the use of the C1s signal as energy calibration at 284.8 eV (see Figure 3). The deconvolution analysis of the XPS spectra shows that the

surface of the double perovskite contains additional oxidation states different from the bulk composition (see Supplementary Figure 5).

The Ba3d core-level spectra in Supplementary Figure 5 show the $3d_{3/2}$ and $3d_{5/2}$ contributions with spin-orbit coupling of 15.3 eV. The deconvolution was carried out with fixed 3:2 intensity ratios to exclude possible Pr MNN overlap at 797 eV and Ba shake-off features on the lower binding energies side of the $Ba3d_{3/2}$ signal. The deconvoluted spectra of the $Ba3d_{3/2}$ peak show two Ba^{II} phases, one that can be related to the bulk perovskite (ca. 779.5 eV) and a different surface phase at ca. 780.8 eV)[2, 3, 4]. The spectra of pristine Ba_2PrIrO_6 look similar to other Ba-containing perovskites (e.g. $BaTiO_3$) with a typical high-energy shoulder at ca. 1.3 eV above the main peak with an intensity ratio of around 0.6[4, 5]. The leaching of barium from the Ba_2PrIrO_6 double perovskite surface is accompanied with a relative decrease in the high-energy component, indicating removal of the surface phase and exposure of the barium-bulk phase. Furthermore, the typical Ba perovskite binding energy shifts approximately 0.3 eV to higher binding energies, indicating the change in environment to a more oxidized character[6]. The oxidizing treatment has a detrimental effect on the Ba content, heavily perturbing both the Ba surface and bulk phase. The ionic character of Ba in addition to its location in the cavity of the PrO_6/IrO_6 octahedra may be the cause of its easier exclusion from the structure.

The Pr3d core-level spectra (see Supplementary Figure 5) consist of two main signals from the ground state 4f configuration with spin-orbit coupling of 20.1 eV and accompanying shoulders and shake-off features resulting from a mixture of final state configurations[7, 8]. The signals were recorded including the O1s Auger line to properly assess the distinct features that indicate a mixture of tri- and tetravalent species. The $Pr3d_{5/2}$ and $Pr3d_{3/2}$ signals of the pristine double perovskite are broad, with a FWHM of 4.7 eV, but the main peaks can be unambiguously fitted with the statistical 3:2 intensity ratio and 20.1 eV spin-orbit coupling, indicating that mainly Pr_{3+} is present[9]. The $3d_{5/2}$ binding energy of 933.8 eV agrees well with the binding energy of praseodymium in a $PrAlO_3$ perovskite lattice[10], approximately 1.0 eV above the value reported for Pr_2O_3[7, 11]. The satellite energy

separation of both signals is large, in the range of the reported values for trivalent Pr in highly electronegative ligand orientation[12]. Satellite spin-orbit coupling of 19.4 eV is lower than the expected (ca. 20.5 eV), and shake-off in the $3d_{3/2}$ region is visible around 958 eV indicating Pr_{4+} contributions, with PrO_2 nature[11, 13]. The main and satellite relative intensity ratios of 0.6 are in the upper limit of trivalent species, and in addition to the abovementioned features, this indicates mixed-valence praseodymium species present in the perovskite structure. The acid treatment induced subtle changes in the spectra of praseodymium, suggesting minor but notable changes in the perovskite surface environment. The binding energy of the main peak shifted 0.3 eV to lower energy, and the width narrowed to FWHM values of 3.9 eV. Moreover, the satellite energy separation decreased and coincides with values for Pr_2O_3. Similarly, the satellite intensity ratio of the $3d_{5/2}$ signal is reduced to values very close to the value in Pr_2O_3[12]. The leaching treatment induces the removal of praseodymium from the perovskite through surface oxidation to form other Pr^{3+} phases. The Ba:Pr ratio is approximately 1:2, which might indicate the formation of a Pr_2BaO_4 spinel surface phase[13].

The Ir4f core-level spectra show two broad signals separated by an extraordinary high saddle between $4f_{5/2}$ and $4f_{7/2}$, indicating two superimposed spectra (see Supplementary Figure 5) [14]. The deconvolution yields two contributions with spin-orbit coupling of 3.0 eV and different FWHM. The high binding energy peak is probably broader as a result of conduction band interaction during the photoemission process[15, 16]. The binding energy values for the $4f_{7/2}$ peak of 62.4 and 64.0 eV are about 0.3 eV higher than reported for iridium compounds with Ir^{IV}, the difference may be attributed to electronic charge transfer with praseodymium in the perovskite[17, 18]. It is striking that the high binding energy peak shifts 0.5 eV towards lower binding energies after the oxidizing treatments, while the low binding energy peak value of 62.4 eV is unchanged. Moreover, the 1:1 relative ratio of the two contributions is maintained after the oxidizing treatment. Taking into account the formal Ir^{IV} valency of the bulk perovskite and the prominent saddle and increasing suspicion of Pr^{III} contributions, the system is likely bivalent with Ir^{IV} and Ir^{V} present in roughly equivalent ratio[1].

The O1s core-level spectra have been resolved with three peaks to distinguish between the contributions of the metal oxides and the surface-adsorbed species (see Supplementary Figure 5). The main peak of the pristine double perovskite is located around 531 eV and is accompanied by a high binding energy shoulder and a low binding energy peak at 529 eV. The main peak is either ascribed directly to the constitutional metal oxide, or more indirectly to oxyhydroxide or metal carbonate signals originating from interactions with the metal oxide surface[4, 11, 15, 17, 18, 19, 20]. The value of 531.1 eV agrees as well with ionic Ba-O-V bonds of non-bridging oxygen in barium vanadophosphate glasses, corresponding to a Ba-O coordination resembling the one in perovskites[21]. The low binding energy peak is ascribed to the O1s signal of a lattice oxygen, more specific from the lattice oxygen bound to Pr^{III} or Pr^{IV} [11, 18, 19]. The O1s metal oxide contribution of perovskite-type $BaTiO_3$ has a similar value of 529.0 eV, which is identical to other Pr-based perovskites such as $PrCoO_3$, and mixed $PrCaMnO_3$, once more indicating overlap of different metal-oxide signals[4]. This lattice oxygen binding energy is somewhat on the low side for Ir^{IV} compounds, however, these values are not reported for perovskite phases[17]. In all abovementioned analyses, the 533 eV peak is assigned to surface contamination from adsorbed water or related hydroxide species. The effect of the oxidizing treatment on the O1s spectra is clear; the main peak further exceeds the high binding energy shoulder and lower binding energy peak. The lattice oxygen contribution is decreased to a minor shoulder, and its binding energy shifts to higher values. Thus, the oxyhydroxide and metal carbonate contribution has increased with respect to the surface contaminants and lattice oxygen, indicating that the surface coverage of the perovskite metal-oxide phase increases. The shift in binding energy is observed as well for $BaTiO_3$ perovskites, where the lattice oxygen signal at 529.0 eV shifted to 529.3 eV after prolonged oxidation times, displaying the electronic interaction of the bulk with the oxidized surface[4]. A shift in binding energy of the lattice oxygen in the case of Pr would imply a reduction from Pr^{IV} to Pr^{III}, which could indicate a surface reorganization with other metals into different mixed-valent phases[13].

Summarizing, the deconvolution analysis of the XPS Ba, Pr, Ir and O core-levels indicate that next to the tetravalent Pr^{IV}/Ir^{IV} valence pair, a surface phase exists containing Pr^{III} and Ir^{V} (see Supplementary Figure 5), however, the chemical nature of these species cannot be confirmed. The oxidizing treatment greatly perturbs the surface of the perovskite, majorly reducing the Ba content to values below that of Pr and Ir. The leaching further enhances the differences in the oxidation state, increasing the Ir^{V} contribution.

Supplementary References

1. Fu WT, Ijdo DJW. On the space group of the double perovskite Ba2PrIrO6. *J. Solid State Chem.* **178**, 1312-1316 (2005).

2. Meyer HM*, et al.* Electronic structures of the YBa2Cu3O7-x surface and its modification by sputtering and adatoms of Ti and Cu. *Phys. Rev. B* **38**, 6500-6512 (1988).

3. Fukuda Y, Nagoshi M, Suzuki T, Namba Y, Syono Y, Tachiki M. Chemical states of Ba in YBa2Cu3O7-d studied by x-ray photoelectron spectroscopy. *Phys. Rev. B* **39**, 11494-11497 (1989).

4. Miot C, Husson E, Proust C, Erre R, Coutures JP. X-ray photoelectron spectroscopy characterization of barium titanate ceramics prepared by the citric route. Residual carbon study. *J. Mater. Res.* **12**, 2388-2392 (1997).

5. Mukhopadhyay SM, Chen TCS. SURFACE CHEMICAL-STATES OF BARIUM-TITANATE - INFLUENCE OF SAMPLE PROCESSING. *J. Mater. Res.* **10**, 1502-1507 (1995).

6. Moulder JF, Chastain J. *Handbook of X-ray Photoelectron Spectroscopy: A Reference Book of Standard Spectra for Identification and Interpretation of XPS Data*. Physical Electronics (1995).

7. Ogasawara H, Kotani A, Potze R, Sawatzky GA, Thole BT. Praseodymium 3d- and 4d-core photoemission spectra of Pr2O3. *Phys. Rev. B* **44**, 5465-5469 (1991).

8. Burroughs P, Hamnett A, Orchard AF, Thornton G. Satellite structure in the X-ray photoelectron spectra of some binary and mixed oxides of lanthanum and cerium. *J. Chem. Soc., Dalton Trans.*, 1686-1698 (1976).

9. Sarma DD, Rao CNR. XPES studies of oxides of second- and third-row transition metals including rare earths. *J. Electron. Spectrosc. Relat. Phenom.* **20**, 25-45 (1980).

10. Kruczek M, Talik E, Pawlak DA, Kołodziejak K, Łukasiewicz T. XPS study of PrAlO3–PrAl11O18 and PrAlO3–Pr2O3 eutectics. *J. Alloys Compd.* **442**, 255-258 (2007).

11. Lutkehoff S, Neumann M, Slebarski A. 3D AND 4D X-RAY-PHOTOELECTRON SPECTRA OF PR UNDER GRADUAL OXIDATION. *Phys. Rev. B* **52**, 13808-13811 (1995).

12. Berthou H, Jørgensen CK, Bonnelle C. Influence of the ligands on 3d photoelectron spectra of the first four lanthanides. *Chem. Phys. Lett.* **38**, 199-206 (1976).

13. Felner I*, et al.* Crystal structure, magnetic properties, x-ray-photoemission-spectroscopy, and specific-heat measurements on Pr2BaO4 and PrBaO3. *Phys. Rev. B* **46**, 9132-9141 (1992).

14. Atanasoska L, Atanasoski R, Trasatti S. XPS and AES study of mixed layers of RuO2 and IrO2. *Vacuum* **40**, 91-94 (1990).

15. Peuckert M. XPS study on thermally and electrochemically prepared oxidic adlayers on iridium. *Surf. Sci.* **144**, 451-464 (1984).

16. Wertheim GK, Guggenheim HJ. Conduction-electron screening in metallic oxides: IrO2. *Phys. Rev. B* **22**, 4680-4683 (1980).

17. Kötz R, Neff H, Stucki S. Anodic Iridium Oxide Films: XPS-Studies of Oxidation State Changes and. *J. Electrochem. Soc.* **131**, 72-77 (1984).

18. Hara M, Asami K, Hashimoto K, Masumoto T. An X-ray photoelectron spectroscopic study of electrocatalytic activity of platinum group metals for chlorine evolution. *Electrochim. Acta* **28**, 1073-1081 (1983).

19. Fissel A, Dąbrowski J, Osten HJ. Photoemission and ab initio theoretical study of interface and film formation during epitaxial growth and annealing of praseodymium oxide on Si(001). *J. Appl. Phys.* **91**, 8986-8991 (2002).

20. Hall HY, Sherwood PMA. X-ray photoelectron spectroscopic studies of the iridium electrode system. *J. Chem. Soc. Faraday Trans.* **80**, 135-152 (1984).

21. Majjane A, Chahine A, Et-tabirou M, Echchahed B, Do T-O, Breen PM. X-ray photoelectron spectroscopy (XPS) and FTIR studies of vanadium barium phosphate glasses. *Mater. Chem. Phys.* **143**, 779-787 (2014).
