# Supplementary material for: Iridium-based double perovskites for efficient water oxidation in acid media
Source: Nat Commun. 2016 Aug 8;7:12363. doi: 10.1038/ncomms12363 (PMC4979062; doi:10.1038/ncomms12363)
Supplement: Peer Review File [file ncomms12363-s2.pdf]

Editorial Note: this manuscript has been previously reviewed at another journal that is not operating a transparent peer review scheme. This document only contains reviewer comments and rebuttal letters for versions considered at Nature Communications.
